# PML, SUMOylation, and Senescence

**DOI:** 10.3389/fonc.2013.00171

**Published:** 2013-07-04

**Authors:** Lisa Ivanschitz, Hugues De Thé, Morgane Le Bras

**Affiliations:** ^1^University Paris Diderot, Sorbonne Paris Cité, Hôpital St. Louis, Paris, France; ^2^INSERM UMR 944, Equipe labellisée par la Ligue Nationale contre le Cancer, Institut Universitaire d’Hématologie, Hôpital St. Louis, Paris, France; ^3^CNRS UMR 7212, Hôpital St. Louis, Paris, France; ^4^Pole Sino-Francais des Sciences du Vivant et de Génomique de l’Hôpital Rui-Jin, Rui-Jin Hospital affiliated with Jiao Tong University, Shanghai, China; ^5^Service de Biochimie, AP-HP, Hôpital St. Louis, Paris, France

**Keywords:** PML, senescence, SUMO, tumor suppression, p53

## Abstract

Since its discovery, 25 years ago, promyelocytic leukemia (PML) has been an enigma. Implicated in the oncogenic PML/RARA fusion, forming elusive intranuclear domains, triggering cell death or senescence, controlled by and perhaps controlling SUMOylation… there are multiple PML-related issues. Here we review the reciprocal interactions between PML, senescence, and SUMOylation, notably in the context of cellular transformation.

## Introduction

Senescence is a permanent cell cycle arrest discovered in human fibroblasts in culture, a process that limits their replicative potential (1). Today, senescence is considered as an important anti-cancer mechanism, probably the first physiological defense against cellular transformation. Moreover, senescent cells that have lost the ability to divide are the underlying mechanism of aging (2, 3). Different types of signals can initiate senescence. Replicative senescence is principally caused by telomere shortening upon repeated cells divisions (4). Beyond a certain point of telomere erosion, the DNA damage response is activated and mediated through the p53 pathway, similarly to double strand breaks. As telomeres cannot be repaired, senescent cells are characterized by persistent reparation foci (5, 6). Premature senescence can also be provoked by signals such as oncogene activation, tumor-suppressor loss, or sustained stress conditions. Retinoblastoma protein (pRB) and p53 suppressor pathways are both major downstream effectors of this stress-induced senescence (7–9).

SUMOylation is a post-translational modification consisting of covalent binding of Small Ubiquitin related Modifier (SUMO) onto a target protein. SUMOs belong to the Ubiquitin-like protein (Ubl) family, and have a very similar three-dimensional structure compared to ubiquitin, while sharing only 20% of sequence identity. In mammals, four SUMO paralogs have been identified. SUMO-1 shares 50% sequence identity with SUMO-2/3, and 86% with SUMO-4. SUMO-2 and 3 are usually pooled together as SUMO-2/3 as they are 95% identical to each other and cannot be separately identified. While SUMO-1 and SUMO-2/3 are ubiquitously expressed, SUMO-4 expression was only found in the liver and consequently its function will not be discussed here (10). SUMOylation is a dynamic process consisting of rapid conjugation and de-conjugation cycles. Target proteins are usually very transiently modified and their SUMOylated forms often constitute only a very small fraction of the total protein pool. SUMO conjugation is performed by specific enzymes in three steps: the SUMO peptides are maturated by SUMO-specific proteases (SENP family), activated by the E1 SUMO activating complex, then transferred onto the E2 SUMO ligase Ubc9 which then conjugates SUMO onto lysine residues of target proteins (11). Substrate interaction and modification can directly be performed by Ubc9 and usually depends on the presence of the short SUMOylation consensus motif (ΨKxD/E) (12, 13). E3 SUMO ligases, which link Ubc9 to the substrate proteins, can also improve target recognition, notably in the absence of SUMOylation consensus sites. The first E3 ligases described were the family of proteins Protein Inhibitor of Activated STAT (PIAS) (14). SUMOs may be removed from their targets by SENP enzymes, the same family of proteases that performs SUMO maturation (15). SUMO conjugation can alter protein–protein interactions, change protein intracellular localization, or directly modify activities resulting in changes in transcription, replication, chromosome segregation, and DNA repair. Importantly, SUMO conjugation has been repeatedly associated to stress response (16).

The promyelocytic leukemia (PML) tumor-suppressor is the key organizer of PML Nuclear Bodies (NBs) (17, 18). These PML-driven structures are characterized by the accumulation of a very large number of nuclear partner proteins. NBs are implicated in multiple cellular processes including virus defense, apoptosis, and senescence. PML NBs are strongly associated with SUMOylation process (19). SUMO was initially described as a PML binding protein (20), SUMO paralogs accumulate in NBs and even NB-biogenesis was proposed to rely on PML SUMOylation (21, 22). PML directly interacts with Ubc9 (23) and may be SUMOylated on three lysine residues. SUMOylation of K160 is essential for PML ability to recruit nuclear partners into NBs (24). SUMOylation state or presence of a SUMO Interacting Motif (SIM) in protein partners were proposed to be the major signals driving their recruitment into NBs (25, 26). Moreover, other key players in the SUMO-conjugation pathway were proposed to accumulate in NBs: several SENP and SUMO E3 ligases, as well as the SUMO-dependent ubiquitin ligase RNF4 (27). Overall, PML NBs are intimately associated with the SUMO pathway, although many functional aspects remain unclear.

Both PML and SUMO overexpression induces senescence. Here, we will discuss the potential links between these two mechanisms.

## PML Induces Senescence

Promyelocytic leukemia NBs have been implicated in various cellular processes. Importantly, PML expression and localization are profoundly changed during oncogenic transformation. First evidence came from the PML/RARA oncoprotein responsible of acute promyelocytic leukemia (APL). PML/RARA oncogene activity relies on its transcriptional repressive activity, but also on its dominant negative action on PML NBs biogenesis (18, 28). Notably, the two active drugs in APL, retinoic acid and arsenic, both allow the reformation of PML NBs, as the result of PML/RARA degradation (29, 30). Moreover, partial or complete loss of PML expression was observed in multiple cancers (31). Several studies have investigated the underlying mechanisms for NBs-loss during tumor progression and have implicated PML degradation, more than loss of *PML* gene expression (32, 33). It was proposed that PML NBs could prevent malignant transformation by promoting senescence (34). Then, loss of PML NBs during tumor progression could reflect loss of this senescence failsafe.

Promyelocytic leukemia plays a key role in senescence induction as demonstrated after stress, DNA damage, oncogene activation, or simply during replicative senescence. Initially, two key observations have directly implicated PML in senescence: Ras-induced senescence is lost in a *pml*^−/−^ context, while conversely, PML overexpression induces premature senescence (35–37). Moreover, PML protein level is increased in replicative or Ras-induced senescent cells. Thus, PML expression is critical for the control of cellular senescence and can be achieved both at transcriptional and post-transcriptional levels. PML promoter contains interferon (IFN) and p53 response elements and can thus be induced by IFN signaling or p53 activation (38, 39). PML up-regulation can be critical in senescence induction, since IFNβ treatment can induce cellular senescence in a PML/p53 dependent manner (40, 41). Genotoxic drug induced senescence was also shown to increase PML transcription through JAK1/STAT1 pathway and IFN production (42, 43). Indeed, senescent cells are characterized and entertained by secretion of multiple cytokines, IFN, and pro-inflammatory factors. This condition has been called senescence-associated secretory phenotype (SASP) (44).

At the protein level, multiple PML degradation pathways were described, several enforced by oncogenic proteins. As an example, proteins such as E6AP ubiquitin ligase, E2F transcription regulator E2FBP1, or Pin1 isomerase promote PML degradation or disrupt NBs formation. Loss or down-regulation of these proteins causes PML stabilization and tumor suppression by senescence induction in human primary fibroblasts or in cancer cells (45–47). Casein Kinase 2 (CK2) and PIAS1 SUMO ligase also regulate PML stability and senescence induction. PIAS1-dependent SUMOylation of PML increases CK2-PML interaction leading to PML phosphorylation at serine 517, ubiquitination, and degradation. Down-regulation of PIAS1 or mutation of serine 517 stabilizes PML and provokes cell cycle arrest (33, 48). Finally, in response to Ras activation, PML is up-regulated through a selective translation initiation depending on PML mRNA 5′UTR and involving the MEK/ERK/mTOR pathway (49). All of these examples demonstrate that PML levels are important in the fine-tuning of senescence induction.

## A Single Isoform Confers PML-Induced Senescence

**Figure 1:**
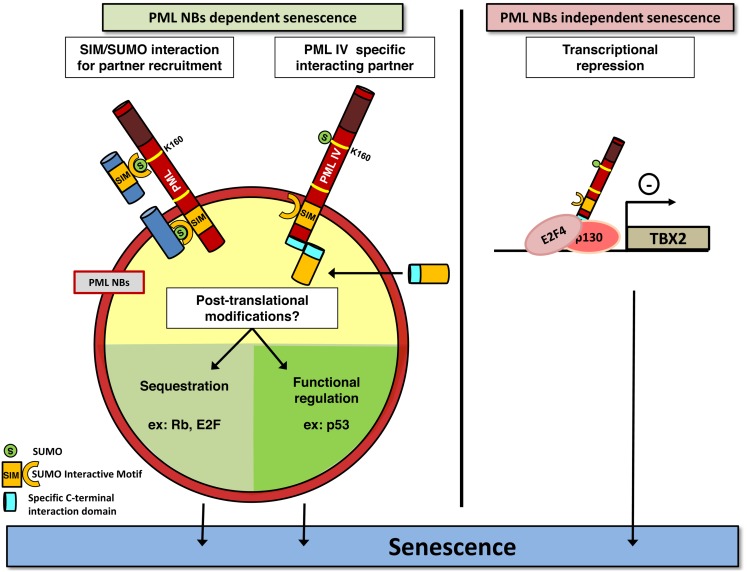
**PML induces senescence**. PML may play a crucial role in senescence induction in a NBs-dependent (left) or independent (right) manner. PML (red) recruits partners (dark blue) through SUMOylation of the key K160 residue. SUMO and SIM motifs are indicated. PML IV could also recruit specific partners into NBs, through a specific binding domain (light blue). This may ultimately favor post-translational modifications of proteins controlling senescence, which converge on E2F and p53. Alternatively, PML IV can act independently of NBs structures, for example, as a transcriptional modulator of specific target genes. As disruption of NBs is a consequence of PML-RARA expression, this model could explain the lack of senescence in APL cells by both the loss of these modification platforms, and loss of PML C-terminal tails and hence defects in specific partners recruitment.

Promyelocytic leukemia is expressed as a number of 3′ splice variants that encode C-terminal distinct isoforms named PML I to PML VII. Only PML IV isoform overexpression induces senescence in primary cells (37). The mechanism by which PML IV isoform elicits this irreversible growth arrest remains controversial and is believed to involve both p53/p21 and p16/Rb pathways. PML IV overexpression stabilizes and activates p53 by inducing its phosphorylation and acetylation (35–37, 50). Since all PML isoforms are able to recruit p53 into NBs, PML IV ability to stabilize p53 is not due to a specific recruitment of p53, but rather of critical partners implicated in p53 modification. During Ras-induced senescence, CBP is recruited to NBs to acetylate p53 (36). Yet, CBP is recruited into NBs by all isoforms and not only PML IV, making it unlikely that CBP is the key limiting factor that allows p53 activation upon PML IV overexpression (37). MORC3 (microrchidia3)-ATPase and the TGF-β negative regulator SnoN both favor senescence, p53 stabilization, and are located in NBs. Moreover, SnoN induced senescence and p53 stabilization are abrogated upon PML extinction (51, 52). Following cellular stress, MOZ acetyltransferase is similarly recruited to NBs, acetylates p53, and enhances p21 expression leading to premature senescence (53). It has also been proposed that specific targeting of HIPK2 into NBs by PML IV would phosphorylate p53, thus facilitating CBP action (54–56). On the contrary, SIRT1 deacetylase is recruited in NBs upon PML IV or Ras expression, to bind and deacetylate p53. Accordingly, overexpression of SIRT1 in MEF cells antagonizes PML IV induced senescence (57). This is also the case for MageA2 overexpression, which interferes with p53 acetylation at NBs and with PML IV-dependent activation of p53 (58). These studies point out the surprising number of p53 regulators recruited to NBs and question the respective importance of each one. The use of HPV oncoprotein E6 and E7, which disrupt p53 or Rb pathway respectively, has rather favored a Rb-dependent mechanism. Indeed, E7 expression completely overcomes PML IV induced senescence while E6 expression has a lesser effect (59, 60). pRB and E2F are sequestered in NBs after enforced PML IV expression thus inhibiting E2F dependent proliferation and DNA repair, leading to p53 activation and senescence (34). This raises the issue of the respective roles of p53 and Rb in enforcing PML-driven senescence (Figure 1).

Altogether, these data demonstrate a front seat role of PML in cellular growth control. PML has clear suppressive function, but whether senescence loss in cancer is due to general PML decrease or only to PML IV specific loss remains an open question. Intriguingly, senescence induction by PML IV was suggested to be a NB-independent function. Indeed, the *Cytomegalovirus* oncoprotein IE1 disrupts NBs, but does not prevent PML IV from triggering senescence (37). In that respect, a last mechanism, which does not require PML NBs formation for PML IV function, was recently described. TBX2 is a transcription factor known to bypass senescence through induction of ARF (CDKN2A locus product p14^ARF^) and p21 repression and to be amplified in several types of cancer (61, 62). TBX2 interacts with PML protein but is not present in NBs (63, 64). However, only PML IV down regulates TBX2 transcription by direct association to its promoter, which leads to senescence. Conversely, TBX2 overexpression inhibits PML IV induced senescence (64). PML IV can also bind to nEGFR, a nuclear form of EGF receptor that exhibits transcriptional activity toward proliferation genes like Cyclin D1 gene, and represses their transcriptional activity. EGF receptor is frequently overexpressed or constitutively activated in non-small cell lung cancer and PML IV isoform expression specifically can repress the growth of these cells (65). Collectively, studies implicating PML IV overexpression have demonstrated a critical role of p53 activation, but the actual molecular mechanism(s) implicated somehow remain(s) under study.

## Sumoylation and Senescence

SUMO overexpression also triggers senescence. Indeed, in HEK and MEF 3T3 cells, overexpression of SUMO-2/3 induces premature senescence (66). In contrast, non-conjugable mutants of SUMO-2/3 failed to induce senescence, suggesting that the increase of SUMO-2/3 conjugation on target proteins is responsible for senescence and not the increase of free SUMO-2/3 in the cell (66). In old rats and senescent fibroblasts in culture, SUMO expression increases with aging and hyper-SUMOylated protein forms become more abundant (67–69). Overexpression of SUMO-1 or SUMO-2 in *Caenorhabditis elegans* led to shortened life span and reproduction disorder (70), while loss of SUMOylation process resulted in severe zebrafish development defects (71) or misdifferentiation and microtumors development of hematopoietic progenitors in *Drosophila* (72). Altogether, these experiments confirm the crucial role of SUMO in development and tumor suppression. SUMO-induced senescence also implicates p53 and pRB, the two major tumor suppressive pathways. Indeed, down-regulation of p53 or pRB by RNA interference counteracts the senescent phenotype induced by SUMO-2/3 overexpression (66). These two proteins are themselves subjected to SUMOylation by both SUMO-1 and SUMO-2/3 paralogs (73–75). Little is known about the functional consequences of SUMO-modified pRB, apart from the fact that pRB SUMOylation mutant seems to be more efficient in E2F repression (75). Two other partners of the pRB pathway have been reported to be SUMOylated. Retinoblastoma binding protein 1 (RBP1) and Stra13 proteins are two transcriptional repressors implicated in cell cycle regulation. In both cases, SUMOylation enhances their repressor activity, leading to cellular arrest and senescence (76, 77). For p53 regulation, SUMOylation clearly acts as a transcriptional modulator, but whether this modification is activating or inhibiting is still under debate, and probably depends on the target genes and the cellular context (78, 79). However a recent *in vitro* study analyzed DNA binding and transcription properties of purified SUMO-1-p53 (80). SUMOylation inhibits binding and transcription of the p21 promoter, and prevents p53 acetylation, arguing that SUMOylation would have an inhibitory effect on p53. In a second study, a gene expression profile was performed by micro-array after expression of wild-type p53 or of a SUMO-1-p53 fusion (81). SUMO-1 modification of p53 can stimulate the expression of a few target genes, but its principal effect seemed to alleviate the trans-repression activity of p53.

## Sumoylation Enzymes Modulate Senescence

Complete loss of SUMOylation is embryonic lethal as shown by E1 activating enzyme knockout in *Drosophila* and E2 ligase Ubc9 knockout in mice or its homolog SAE2 in plants (82–84). Partial loss of SUMO machinery enzymes activity or expression level, including SENP proteases and E3 ligases, also triggers many cellular defects. Some situations linking SENP1 and SENP2 repression to senescent phenotype have been observed. Acute repression of SENP1 by shRNA retroviral infection induces Human Foreskin Fibroblast (HFF) cells premature senescence correlated with a strong accumulation of high molecular weight SUMO-1 conjugates (85) located in PML NBs. This phenotype has also been described with SENP2 and SENP7 repression and SUMO-2/3 consecutive accumulation. In another study, the silencing of the nuclear pore protein Tpr is responsible for senescence of HeLa and U2OS cells through reduction of SENP2 level (86). Interestingly, the Tpr-induced senescence is reversed by SUMO-1 siRNA extinction, emphasizing the importance of SUMO pathway in senescence. It worth mentioning that SENP depletion induced senescence is dependent on p53 activity, and it would be interesting to evaluate the role of SUMOylated p53 in this context. Overexpression of the SUMO E3 ligase PIASy has been associated with senescence induction in human fibroblasts (87). Direct interaction of PIASy with pRB and p53 promotes pRB transcriptional repression of E2F target genes and p53 SUMOylation and transcriptional activation (87). The ubiquitin ligase TRIM32, mutated in limb-girdle muscular dystrophy 2H disease (LGMD2H), normally targets PIASy for degradation. TRIM32 deficient mice harbor PIASy and SUMOylated proteins accumulation leading to premature senescence of muscle satellite cells (88). Nevertheless, PIASy induced senescence may strongly depend on cellular context since two other studies point out its proliferative effect and cell cycle re-entry capacity (89, 90).

ARF, a key regulator of p53 pathway, was also shown to play a role in SUMOylation. If ARF tumor-suppressor activity is mainly mediated through p53, ARF also exerts p53-independent functions since its overexpression in p53-null MEFs triggers proliferation arrest (91). One of these p53-independent roles could link ARF to SUMO modifications, as ARF overexpression induces an increase of global SUMOylation in 293T, U2OS, and 8054 human colon cancer cells expressing His tagged SUMO-1. ARF also promotes the SUMOylation of various specific targets, among them Hdm2, p53, p63, NPM, E2F1, Werners Helicase (WRN), and others (92–96). To note, ARF-induced SUMOylation of Hdm2 seems not to interfere with p53 signaling, and would be part of another cellular process (95). Finally, ARF was shown to specifically interact with the E2 SUMO ligase Ubc9 making the link with the target proteins, thus arguing that ARF may be an effective E3 SUMO ligase (94).

## A Connection between PML and SUMOs for Senescence Control?

**Figure 2:**
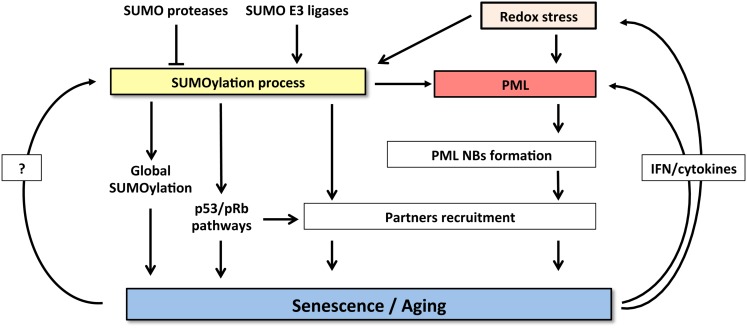
**Crosstalk between SUMOylation machinery and PML pathway controls senescence induction**. SUMOylation process and PML NBs functions are highly cross-connected. Increase of both SUMO and PML levels induces senescence. SUMOylation enzymes regulate NBs formation and partners’ recruitment. Conversely, NBs could potentiate SUMOylation process and partners modification. Finally, senescent cells express specific cytokines (IFNs or IL-6) that, in a positive feedback loop, enhance PML expression and also induce oxidative stress further enforcing NBs formation.

Taken the key roles of SUMO and PML in promoting senescence and the profound effects of SUMOs on PML biology and NB-organization, it is tempting to propose that the two pathways could be connected. Many reciprocal interactions exist between SUMO and PML. Global increase of SUMOylated proteins provoked by SENP1 repression induces NBs enlargement (85). PML IV was suggested to behave as an E3 SUMO ligase for p53 and HDM2 (97). The other PML isoforms, ineffective at inducing senescence, do not enhance p53 SUMOylation, suggesting that this modification may contribute to the senescent phenotype. PML overexpression could increase recruitment of SUMO machinery and consequently enhance global SUMOylation, provoking premature senescence. Indeed, NBs concentrate a large amount of SUMOylated proteins compared to other cellular compartments (19). Then, the tumorigenic switch observed in cancer after loss of PML NBs might either be explained by primary alterations of SUMOylation homeostasis leading to secondary defects in NB-assembly or, alternatively, to altered global SUMOylation as a consequence of PML loss (Figure 2).

Finally, SUMO modification participates in cellular senescence through modulation of oxidative stress responses (Figure 2). In this context, it has been shown that SUMOylation of HIPK2 (98) or key enzyme such as NADPH oxidase 2 (99) can inhibit their functions and consequently may control redox status (Figure 2). Interestingly, the fact that PML is sensitive to oxidative stress, through the formation of disulfide bridges that trigger NB-biogenesis (100), could contribute to stress-induced SUMOylation and cooperate to mediate senescence. Taken the rising importance of senescence, SUMOs, and PML in cancer genesis and also therapy response, exciting future developments are to be expected in this rapidly moving field.

## Conflict of Interest Statement

The authors declare that the research was conducted in the absence of any commercial or financial relationships that could be construed as a potential conflict of interest.
